# Response to organic cultivation of heirloom *Capsicum* peppers: Variation in the level of bioactive compounds and effect of ripening

**DOI:** 10.1371/journal.pone.0207888

**Published:** 2018-11-21

**Authors:** Ana M. Ribes-Moya, María D. Raigón, Estela Moreno-Peris, Ana Fita, Adrián Rodríguez-Burruezo

**Affiliations:** Instituto COMAV. Edificio 8E, acceso J. Universitat Politècnica de València, Valencia, Spain; USDA/ARS, UNITED STATES

## Abstract

Peppers (*Capsicum spp*.) are one of the most important vegetables and their double use (vegetable or spice) and two commercial stages (unripe and fully ripe) contributed to their use in many recipes and fast diffusion from America. Nowadays, Spain is a center of diversity for *C*. *annuum*, with many landraces, offering a great opportunity for adaptation to organic cultivation. Furthermore, *Capsicum* peppers contain many bioactive compounds, essential to provide high added-value to these cultivars, especially for organic markets, although knowledge about the effect of organic cultivation on *Capsicum* fruit quality is still scarce. Here, 37 accessions of Spanish landraces and foreign materials from *C*. *annuum* and other species were grown under organic and conventional conditions and evaluated for ascorbic acid (AAC), total phenolics (TP) and total red and yellow/orange carotenoids, considering both ripening stages. A large genotypic variation was found within each ripening stage and growing condition for the studied traits. Also, both stages showed high levels, although fully ripe fruits were the richest. Organic conditions enabled higher levels in fully ripe fruits of AAC and TP on average (135 vs 117 mg·100 g^-1^ and 232 vs 206 mg·100 g^-1^) and in most accessions, although the genotype×growing conditions interaction also contributed, but at lower extent, to the observed variation. Significant genotype×ripening stage and growing conditions×ripening stage interactions were also found, suggesting that the magnitude of the increase with ripening depends on the accession and growing conditions. By contrast, there were no differences between growing conditions for carotenoids and differences were mainly due to the genotype factor. Finally, the large genotypic variation and favourable organic conditions allowed identifying several materials from different types and uses with very high levels of bioactive compounds for organic cultivation, in both ripening stages but particularly at fully ripe stage (>500 mg·100 g^-1^).

## Introduction

The increase of human population and changes in their needs are causing a considerable impact on the use of land for cultivation and drives to an unsustainable use of agricultural inputs [1]. Furthermore, human selection practices, unsustainable agricultural and industrial activities, and spreading of urban areas have brought to displacement and readjustment of natural ecosystems [2]. All these facts are driving regional, national, and international institutions to consider a change in the management of resources and inputs towards a more efficient use and exploitation [3,4]. For these reasons, the efforts in more sustainable and environment-friendly agricultural practices are increasing. Furthermore, organic agriculture is expanding in the last years, offering farmers an alternative of diversification of their production. In addition, Europe has increased the acreage for organic agriculture, from 6.8 million hectares in 2005 to 13.5 million hectares in 2016 [5]. This is mainly due to a growth in the demand of consumers, who require products coming from more sustainable production systems [6,7].

Nowadays, most cultivars used in organic production are modern F1 materials, with a narrow genetic pool, and initially developed for conventional high-input conditions. By contrast, heirlooms, ecotypes and local varieties encompass a wide genetic diversity and evolved and were bred under low input conditions, similar to those of the current organic agriculture. Also, consumers are increasing the demand in products with “the taste of the past” of ancient landraces. Furthermore, the wide diversity encompassed by traditional cultivars offers the opportunity to select high added value materials on the basis of their composition, which is also another highly demanded trait from consumers. Finally, promoting the use of traditional landraces and ecotypes under organic cultivation contributes to mitigate the genetic erosion of agrodiversity in terms of environmental and ecological characteristics [8].

In this regard, the fruits from *Capsicum* species, known as peppers or chillies, are grown worldwide with a harvested area of 1.7 and 1.9 million ha of dry chillies and fresh peppers in 2014, respectively [9]. Native from America, they encompass a huge genetic variation and there are five cultivated species, including the common pepper *C*. *annuum* and its relatives, members of the *annuum* complex, *C*. *chinense* (e.g. Habanero, Jolokia types) and *C*. *frutescens* (e.g. Tabasco types, bird peppers). In addition, other species like *C*. *baccatum* or South American *ají* and *C*. *pubescens* or *rocoto* are profusely cultivated and consumed in the Andean region [10,11]. *C*. *annuum* is spread worldwide and has become the most important species, with a plethora of varietal types adapted to a wide range of agroclimatic conditions [12]. *Capsicum* peppers, both unripe and fully ripe stages, have a range of culinary uses as vegetable and/or spice, colorant or preservative agents, as well as medicinal, cosmetic and ornamental uses [13,14]. Moreover, Spain is a relevant center of diversity, especially for *C*. *annuum* since it was introduced in the 15^th^ century from America. The diversity of agroclimatic conditions and a process of adaptation and traditional breeding performed by generations of farmers arose many ecotypes and landraces. In fact, *C*. *annuum* is the vegetable with the highest number of protected designations of origin (PDOs) and protected geographical indications (PGIs) in Spain [8].

As already mentioned, these materials could be exploited by their environment adaptation and quality. In this regard, *Capsicum* peppers have been reported to have the highest antioxidant properties among common-use vegetables as a result of their high levels of bioactive compounds, e.g. ascorbic acid, phenolics and carotenoids [15–17]. Ascorbic acid (i.e. vitamin C, mainly L-ascorbic), is one of the most powerful antioxidants [18] and appears at high levels in *Capsicum* pods, increasing with ripening [19] and considerably higher than other fruits such as orange or kiwifruit [20]. Phenolics are secondary metabolites with very diverse roles: defence against pests and stresses, contribution to fruit flavour and colour, growth regulation, etc. [21,22]. Flavonoids (e.g. quercetin and luteolin) are the most abundant group in *Capsicum* fruits and significant antitumoral, antioxidant and antiviral activities have been attributed to these metabolites [23–25]. Finally, the diversity of colours in the fully ripe fruits of *Capsicum* is due to a combination of up to more than thirty carotenoids. They contribute as food colorants, but also as protectors against photooxidation and providing health benefits as antioxidants, vitamin A precursors or antitumoral agents [26–30]. Carotenoids are mainly present in the fully ripe fruits of *Capsicum*, while their level are comparatively negligible (or even nil) in unripe fruits [31]. Therefore, these traits are of paramount importance for breeding peppers and, at the same time, for promoting the use of traditional varieties of *C*. *annuum* by increasing their added value in the frame of organic production and markets.

Despite there are some interesting reports on the levels of bioactive compounds in *C*. *annuum* under organic practices, most of them were based on a scarce or nil varietal diversity and/or only considered one ripening stage [32–34]. Thus, the knowledge about the range of responses expected from the great variation available in the traditional varieties of this crop, as well as the opportunities for exploiting the genotype-by-environment interaction at each ripening stage, remains almost nil. The objective of the present work is to assess, in a comprehensive collection of *C*. *annuum* and other exotic Capsicums, the response of different genotypes to organic practices in comparison to conventional practices in terms of the levels of their main bioactive factors, i.e. ascorbic acid, phenolics, and carotenoids, considering also the two main commercial stages, unripe and fully ripe. The effect of ripening stage and growing conditions, as well as the interactions of cultivars (genotype) with these factors and the opportunities of selecting materials for specific growing conditions are also discussed. To our knowledge, this is the first comprehensive comparative study in *Capsicum* for adaptation to organic conditions by exploiting their variation in bioactive compounds.

## Materials and methods

### Plant material

A collection of thirty-seven accessions of *Capsicum* was evaluated in this work, encompassing a wide diversity of varietal types and origins, including many Spanish traditional varieties or ecotypes from PDOs and PGIs and several peppers and chillies from other countries (Table 1, S1 and S2 Figs). This collection mainly included *C*. *annuum* accessions (30 accessions), but also included *C*. *chinense* (4), *C*. *frutescens* (1) and South American ají *C*. *baccatum* (2).

**Table 1:** List of accessions used, with local name, origin and fruit traits, and years of evaluation in the experiment.

Cultivar/accession	Origin	Colour	Mesocarp	Shape (Pochard type)	Length/width (mm)	Weight (g)	Year(s)
*C*. *annuum*							
Bell peppers							
1. Bierzo	Cons. Reg. PGI Pimiento Asado Bierzo. Carracedelo, Leon (Spain)	Red	Thick, fleshy	Conical/heart (P)	104/94	100–200	2015–2016
2. CW Aguila F_1_	Syngenta Seeds Spain	Pale red	Thick, fleshy	Squared (A1-A2)	112/97	200–300	2015
3. CW breeding line	COMAV Institute. Valencia (Spain)	Red	Thick, fleshy	Squared (A1-A2)	119/105	200–300	2015
4. Cuneo	S. Lanteri. Torino, Piedmont (Italy)	Yellow	Thick, fleshy	Squared (A2)	115/109	>250	2015–2016
5. Najerano	Cons. Reg. PGI Pimiento Riojano. Logroño, La Rioja (Spain)	Red	Thick, fleshy	Triangular elongated (C3)	146/68	150–250	2015
6. Pimiento Valenciano	COMAV Institute. Valencia (Spain)	Red	Thick, fleshy	(Bell) Elongated (B1)	162/73	>250	2015
Other *C*. *annuum*							
7. Ancho	Puebla (Mexico)	Deep red	Medium	Triangular (C4)	70/45	25–50	2015
8. Arnoia	PGI Pemento da Arnoia. Orense, Galicia (Spain)	Red	Medium	Elongated (B3)	75/48	25–50	2015
9. Berbere	Ethiopia. Reimer´s Seeds Co.	Red	Thin, high dry matter	Elongated (B4)	68/18	<10	2015
10. Bola	Cons. Reg. PDO Pimentón Murcia. Totana, Murcia (Spain)	Deep red	Thin, high dry matter	Round (N)	35/41	10–25	2015–2016
11. Chile de Arbol	Mexico. Reimer´s Seeds Co.	Red	Thin, high dry matter	Very elongated (C1)	75/10	<10	2015
12. Chimayo	Chimayo, New Mexico (USA)	Red	Medium	Elongated (C2-C3)	141/32	25–50	2015
13. Di Senise	S. Lanteri. PGI Peperone di Senise. Senise, Potenza (Italy)	Deep red	Thin, high dry matter	Elongated (C2)	152/37	25–50	2015–2016
14. Doux Long des Landes	Françoise Jourdan. INRA Geves (France)	Red	Medium	Very elongated (C1)	156/23	25–75	2015–2016
15. Espelette	Françoise Jourdan. Type PGI Piment d´Espellette. INRA Geves (France)	Deep red	Thin, high dry matter	Elongated (C3)	138/29	25–50	2015–2016
16. Gernika	I. Ruiz de Galarreta. PGI Gernikako Piperra. NEIKER, Euskadi (Spain)	Deep red	Thin, high dry matter	Elongated (C2)	84/32	25–50	2015–2016
17. Guindilla Ibarra	I. Ruiz de Galarreta. EUSKO-Quality Label. NEIKER, Euskadi (Spain)	Red	Thin, high dry matter	Very elongated (C1)	139/11	<10	2015–2016
18. Jalapa F_1_	P.W. Bosland. NMSU, Las Cruces, New Mexico (USA)	Pale red	Thick, fleshy	Elongated (B4)	75/21	10–25	2015
19. Jalapeno Candelaria	P.W. Bosland. NMSU, Las Cruces, New Mexico (USA)	Pale red	Thick, fleshy	Elongated (B4)	68/22	10–25	2015
20. Jalapeno Espinalteco	P.W. Bosland. NMSU, Las Cruces, New Mexico (USA)	Pale red	Thick, fleshy	Elongated (B4)	78/22	10–25	2015
21. Mojo Palmero	Reserva Mundial de la Biosfera. La Palma, Canary Islands (Spain)	Deep red	Thin, high dry matter	Elongated (C2)	56/21	10–25	2015–2016
22. Numex 6–4	P.W. Bosland. NMSU, Las Cruces, New Mexico (USA)	Pale red	Medium	Elongated (C2)	225/36	50–200	2015
23. Numex Big Jim	P.W. Bosland. NMSU, Las Cruces, New Mexico (USA)	Red	Medium	Elongated (C2)	215/34	50–200	2015–2016
24. Numex Conquistador	P.W. Bosland. NMSU, Las Cruces, New Mexico (USA)	Red	Medium	Elongated (C2)	231/32	50–200	2015
25. Padron	Cons. Reg. PDO Pemento Herbón. Coruña/Pontevedra, Galicia (Spain)	Red	Thin, high dry matter	Elongated (B4)	65/26	<10	2015–2016
26. Pasilla	Mexico. Reimer´s Seeds Co.	Brown	Thin, high dry matter	Very elongated (C1)	210/25	10–25	2015–2016
27. Petit Marsellais	Françoise Jourdan. INRA Geves (France)	Orange	Thin, high dry matter	Elongated (B2)	75/42	10–25	2015
28. Piquillo	Cons. Reg. PDO Pimiento Piquillo de Lodosa. Lodosa, Navarra (Spain)	Deep red	Medium	Triangular (C4)	94/48	50–100	2015–2016
29. Serrano Criollo	COMAV Institute. Valencia (Spain)	Red	Thin, high dry matter	Elongated (B4)	38/14	<10	2015
30. Serrano	Mexico. Reimer´s Seeds Co.	Red	Medium	Elongated (B4)	35/16	<10	2015–2016
Other *Capsicum* species							
31. BOL-37R (*C*. *baccatum*)	Sillane. Chuquisaca (Bolivia)	Red	Medium	Elongated	105/26	5–15	2015
32. BOL-58 (*C*. *baccatum*)	Cochabamba. Cochabamba (Bolivia)	Deep red	Thin, high dry	Elongated	70/15	5–10	2015–2016
33. Aji dulce (*C*. *chinense*)	Caracas (Venezuela)	Pale red	Thin, high dry matter	Flattened	20/35	5–10	2015
34. ECU-973 (*C*. *chinense*)	El Chaco, Napo (Ecuador)	Red	Thin, high dry matter	Elongated	44/12	5–10	2015
35. ECU-994 (*C*. *chinense*)	Archidona, Napo (Ecuador)	Red	Thin, high dry matter	Triangular	42/15	5–10	2015–2016
36. PI-152225 (*C*. *chinense*)	COMAV Institute. From USDA (USA)	Deep red	Thin, high dry matter	Elongated	40/11	5–10	2015
37. BOL-144 (*C*. *frutescens*)	Yapacani. Santa Cruz (Bolivia)	Red	Thin, high dry matter	Very elongated	26/6	1–3	2015

### Growing conditions

Plants materials were grown open field in two growing conditions, organic and conventional, in the spring-summer growing season of years 2015 and 2016. To work with large numbers of accessions, individuals and fruit analyses is tedious and time-consuming and also involves several technical and logistics difficulties. For this reason, our experiment and materials were conducted in two years and a group of 16 accessions were evaluated in both years as controls of the year effect (Table 1). In both growing conditions plants were transplanted at the 4–5 true leave stage, in April 2015 and 2016 and at planting frame 1×0.5 m. Both organic and conventional experimental plots were located in the area of Sagunto (northern Valencia city, Spain). The organic plot was located in the *Marxal dels Moros* protected area (UTM coordinates X: 734494.88, Y: 4390434.86), while the plot managed under conventional conditions (control) was close to the organic plot to get the same conditions in climate, water, and irrigation time, and similar soil main properties (see S1 Table) (UTM coordinates X: 732900.40/ Y: 4391754.37). Plants were irrigated by surface irrigation every 8–10 days, depending on the evapotranspiration, from the irrigation ditch that bordered the plots. In this way, both growing systems differed mainly in management and historical soil conditions.

Both plots were managed by technicians and farmers from the *Unió de Llauradors i Ramaders* (LA UNIÓ), one of the most relevant farmers association in organic production in the Region of Valencia, following their usual organic or conventional practices. The organic management was based in a soil with rotation of crops every four years. Also, fertilization was based on organic sheep manure (4 kg/m^2^) at the beginning of the season. External treatments for pest control were not necessary as natural predators maintained the microfauna balance. Adventitious plants were controlled by hand tools monthly and three thermal applications (i.e. gas burners) per season, applied on emerging plantlets. The conventional management included fertilization based on one application of vegetable humus (4 kg/m^2^) and one application of a mix of nitrogen, phosphorus and potassium (15-15-15) (50 g/m^2^) before transplanting. After transplanting calcium nitrate was applied at three times (one at 20 g/L and two at 10 g/L) and iron chelate was applied once at a dose of 3 kg/1000 m^2^. Pest and diseases were controlled by applying chlorpyrifos (48%, EC) and abamectin (1.8%, EC) as pesticides, combined with copper oxychloride (58.8% WP) as fungicide. Copper oxychloride (100 cc) combined with chlorpyrifos (50 cc) in 20 L of water were applied six times along the growing season, while copper oxychloride (100 cc) combined with abamectin (30 cc) in 20 L of water were applied three times. Adventitious plants were controlled in the same way as described for the organic plot.

### Experimental design and sample preparation

For the design of both plots a random distribution model was arranged. Each accession was represented within each growing system by ten plants distributed in two blocks of five plants randomly distributed the plot. In this way, the position effect in each experimental plot was mitigated and each variety was subject to a generalized environmental distortion within the plot, randomly distributing the position effect. Each plot was surrounded by a bordure of plants to prevent the border effect.

A total of five samples per accession × growing condition × ripening stage combination were prepared per year. Each sample was prepared with fruits from the two plants of each block (n = 5) and therefore the 10 plants per accession were sampled. Fruits were harvested twice per week at both stages of commercial maturity: i) unripe, i.e. final size, firm fruit but still green, ii) fully ripe, i.e. final size, fully covered of carotenoids and still firm) from the end of June to the end of October. Fruits were harvested at both stages when fitted these criteria according to our experience managing diverse *Capsicum* germplasm and the commercial expertise of LA UNIÓ staff. Each sample was divided into two subsamples i) 15 g of fresh fruits were used for the ascorbic acid analysis and ii) 30 g of fresh fruits were lyophilized and preserved in dark and dry conditions for the subsequent analysis of phenolics and carotenoids. Due to their very low or nil content in unripe fruits, the analyses of carotenoids were only considered in fully ripe samples, while ascorbic acid and phenolics were estimated in both commercial stages. Thus, a total of 1060 subsamples were prepared (530 unripe stage and 530 from fully ripe stage) for each trait under study in the two years of the experiment, and a total of 2650 analyses were done (1060 unripe stage and 1590 fully ripe stage).

### Analytical methods

Ascorbic acid content (AAC), total phenolics (TP) and total red carotenoids and yellow/orange carotenoids (C_R_ and C_Y/O_) were estimated following different spectrophotometrical methods with high accuracy and adapted to peppers, which enable to process large amounts of fresh and dried samples, essential for breeders in order to evaluate and compare accurately large collections of cultivars [35,36].

Ascorbic acid content (ACC) was determined reflectometrically, based on the reduction of yellow molybdophosphoric acid to phosphomolybdene blue. The equipment used was a reflectometer *RQflex plus* and a test of ascorbic acid *Reflectoquant* (Merck, Darmstadt, Germany). Samples of 15 g of fresh fruit pulp were homogenized with an industrial blender by adding distilled water to facilitate blending. After the homogenization the liquid was filtered with a mesh (0.5 mm) in a 100 mL test tube and it was filled with distilled water up to 50 mL for unripe fruits and 100 mL for fully ripe fruits. The results were expressed in mg of ascorbic acid per 100 g of fresh weight, according to the following equation:

$$\mathrm{AAC}\;(\mathrm{mg}∙100g^{-1})=\frac{\mathrm{Measure}\;\mathrm{value}\;(\mathrm{mg}∙L^{-1})\times \mathrm{Volume}\;(\mathrm{mL})}{10\times \mathrm{fresh}\;\mathrm{fruit}\;\mathrm{weight}\;(g)}$$

Total phenolics (TP) were estimated according to Folin-Ciocalteu methodology, based on a colorimetric reaction quantified by measuring absorbance at 750 nm, and results were referred according to a standard curve of chlorogenic acid as reported by [36]. This analysis is used to determinate total phenolic antioxidants by measuring the reducing capacity [36,37]. Prior to the analysis, each sample was submitted to extraction according to the following protocol: 125 mg of lyophilized fruit were placed in 15 mL Falcon centrifuge tubes and 5 mL of extraction solution (700 mL acetone, 5 mL glacial acetic acid and 295 mL Milli-Q® water) were added. Then samples were incubated and stirred for 24 hours at room temperature. Afterwards, the samples were centrifuged (3500 rpm for 3 min) and 1.5 mL of the supernatant was collected in a microcentrifuge tube and stored at -80°C until analysis. For the analyses, samples were centrifuged again (10000 rpm for 5 min) and to 65 μL of the sample were added 500 μL of Folin-Ciocalteu reagent (diluted with water at 10%, v/v) and were incubated for 5 min at room temperature. After that, 500 μL of a saturated sodium carbonate solution (60 g/L) were added and the mixture was incubated for 90 min in darkness at room temperature. Then, an aliquot (200 μL) of each sample or standards was placed in a 96-well microplate, and absorbance was measured at 750 nm using a microplate reader (Bio-Rad iMark^TM^, Herts, England). Based on the dry matter content of each sample, results were expressed as mg of chlorogenic acid per 100 g of fresh weight.

Determination of carotenoids was carried out according to a modification of the spectrophotometric method developed by [38], which estimates accurately the sum of the main red (TC_R_ = capsanthin + capsorubin) and yellow-orange (TC_Y/O_ = β-carotene + β-criptoxanthin + lutein + violaxanthin + zeaxanthin) isochromic carotenoid fractions in *Capsicum* fruits. The extraction was made from 100 mg of lyophilized samples in a 100 mL Erlenmeyer flask with 20 mL of acetone and shaking for 1 hour in darkness at room temperature. Then the sample was filtered with filter paper (Watman n°2) and the filtered dilution was filled up to 25 mL in a volumetric flask with acetone. A sample of 3 ml of this dilution was introduced in glass cells with an optical path length of 10 mm and the absorbance of the extract was measured at 472 and 508 nm using a visible-UV spectrophotometer (UviLine 9400, SI Analytics, Weilheim, Germany) and comparing to acetone as blank. Lambert-Beer equation for multicomponent mixtures was applied according to the following equations:

$${\mathrm{TC}}_{R}\;(\mu g∙{\mathrm{mL}}^{-1})=\frac{\Delta_{508}\;\mathrm{nm}\times 2144.0-\Delta_{472}\;\mathrm{nm}\times 403.3}{270.9}$$

$${\mathrm{TC}}_{Y/O}\;(\mu g∙{\mathrm{mL}}^{-1})=\frac{\Delta_{472}\;\mathrm{nm}\times 1724.3-\Delta_{508}\;\mathrm{nm}\times 2450.1}{270.9}$$

Finally, as for TP, carotenoid content was expressed in mg per 100 g fresh weight.

### Statistical analysis

The values of the 16 accessions evaluated both years were used to estimate and correct the year effect. The significance of the year effect was estimated by ANOVA and the data were corrected by subtracting the corresponding mean year deviation (D_y_) to the data from all accessions. Mean year deviation was estimated as follows: D_y_ = μy—μ_T_, where D_y_ is the mean deviation of year y, μ_y_ is the total mean of the 16 accessions in year y and μ_T_ is the total mean of both years. The results of two years were expressed as a mean for each combination of genotype × ripening stage × growing system. Normality was evaluated by W of Shapiro-Wilks test and homocedasticity was analysed by Bartlett and Levene’s tests, both performed by Statgraphics Centurion XVI software (StatPoint Technologies, Inc; Warrenton, Virginia, USA).

Required transformations were performed to adapt the data to a normal distribution model [39]. Then the analysis of variance ANOVA of main factors genotype, growing system, ripening stage and their interactions were carried out, in both original and transformed data, in order to verify the statistical interpretation. The linear model used for the general ANOVA was X_ijkl_ = μ + a_i_ + b_j_ +c_k_ + (α × β)_ij_ + (α × γ)_ik_ + (β × γ)_jk_ + e_ijk(l)_, where X_ijkl_ is the value for fruit sample l of genotype i, growing system j and ripening stage k; μ is the general mean; a_i_ is the effect of genotype i; b_j_ is the effect of growing system j; c_k_ is the effect of ripening stage k; (α × β)_ij_ is the interaction between genotype i and growing system j; (α × γ)_ik_ is the interaction between genotype i and ripening stage k; (β × γ)_jk_ is the interaction between growing system j and ripening stage k and e_ijk(l)_ is the effect of fruit sample l from the combination of genotype i, growing system j and ripening stage k (i.e. the error term). Also specific ANOVA considering separately unripe and fully ripe stages were performed and the linear model used was X_ijk_ = μ + a_i_ + b_j_ + (α × β)_ij_ + e_ij(k)_, where X_ijk_ is the value in one specific ripening stage for fruit sample k of genotype i and growing system j; μ is the general mean; a_i_ is the effect of genotype i; b_j_ is the effect of growing system j; (α × β)_ij_ is the interaction between genotype i and growing system j and e_ij(k)_ is the effect of fruit sample k from the combination of genotype i and growing system j. No differences were found between the original and transformed data in terms of the results of the ANOVA and, therefore, our results were showed and discussed on the basis of the original nontransformed data to facilitate the understanding.

Genotype×environment (i.e. accession×growing system) interactions were studied by means of regression analysis [35]. Regression coefficients (β) of each variety were calculated from the average contribution of each growing system according to the formula β_i_ = (μ_ij_−μ_ijk_) / environmental mean = (μ_ij_−μ_ijk_) / (μ_j_−μ_jk_); where β_i_ is the regression coefficient value for each specific trait and ripening stage for genotype i; μ_ij_ is the mean of genotype i in growing system j; μ_ijk_ is the mean of genotype i for both growing systems j and k, μ_j_ is the mean for all the genotypes in growing system j, μ_jk_ is the mean for all the genotypes in both growing systems j and k. The genotypes with β values that did not differ significantly from 0 were considered stable against the growing system effect [40], i.e. they did not show significant differences between the growing systems for the trait under study.

## Results and discussion

### Analysis of variance in ascorbic acid, phenolics and carotenoids

The year effect had significant contribution to the variation observed in all traits, which indicates that all the traits studied experienced significant changes in their levels from one year to the other, as can be expected in open field trials in agriculture. For that reason and in order to use all the data from both years, the values were corrected considering the year effect. The general ANOVA revealed that the effect of the genotype and particularly the ripening stage were significant and contributed considerably to the variation of AAC and TP according to the magnitude of their mean squares (Table 2). By contrast, the effect of the growing system was only significant for the observed variation in ACC. In addition, the interactions between main factors were also significant and, particularly, those including the ripening stage (Table 2).

**Table 2:** General ANOVA for the content of ascorbic acid (AAC) and total phenolics (TP) and specific ANOVA corresponding to each ripening stage for AAC, TP, total red carotenoids (TCR), and total yellow/orange carotenoids (TCY/O).

Effect	AAC		TP		TC_R_		TC_Y/O_	
	df^a^	MS^b^	df	MS	df	MS	df	MS
General ANOVA								
Main effect								
Genotype (G)	36	15921***	36	73148***	-^c^		-	
Growing system (S)	1	11118***	1	2864^NS^	-		-	
Ripening stage (R)	1	1570610***	1	844879***	-		-	
Interactions								
G x S	36	4312***	36	10445***	-		-	
G x R	36	7932***	36	28606***	-		-	
S x R	1	21654***	1	179547***	-		-	
Error		467		2544	-		-	
Unripe stage ANOVA								
Main effect								
Genotype (G)	36	6775***	36	63183***	-		-	
Growing system (S)	1	410^NS^	1	55864***	-		-	
Interactions								
G x S	36	3501***	36	8394***	-		-	
Error		283		882	-		-	
Fully ripe stage ANOVA								
Main effect								
Genotype (G)	36	15836***	36	37459***	36	74573***	36	25853***
Growing system (S)	1	24720***	1	102713***	1	4369^NS^	1	43^NS^
Interactions								
G x S	36	2484***	36	12353***	36	2945^NS^	36	318^NS^
Error		601		4097		2942		963

^a^degrees of freedom

^b^mean square

^c^Carotenoids were evaluated only at fully ripe stage.

^NS^, *, ** and *** indicate not significant for a probability p >0.05 and significant for p <0.05, 0.01 and 0.001, respectively, according to the statistical F ratio.

Furthermore, mean square values showed a remarkable contribution of the ripening stage in both AAC and TP, not only as a main factor but also as part of interactions with the other factors, which could be biasing the real magnitude of the contribution of the genotype and the growing system, specially the latter. For this reason, further ANOVA considering separately data from unripe fruits and from fully ripe fruits were performed.

Thus, the results of the ANOVAs for each ripening stage showed a significant contribution of both the genotype and growing system factors at both stages, with the only exception of AAC at the unripe stage (Table 2). Furthermore, the contribution of the growing system factor, on the basis of mean square values, increased considerably in comparison to that of the general ANOVA, confirming that the effects of the interactions of the ripening stage with both the genotype and the growing system hidden the real effect of the main factor growing system. Also, the genotype × growing system interaction was found to be significant for AAC and TP at both ripening stages, suggesting that such interactions could be exploited for selection in these traits. On the basis of these results, the descriptive study of the results will be then displayed in the next sections considering separately both the unripe and fully ripe fruits.

Finally, the analysis of variance for carotenoids (only at fully ripe stage) showed a high significant contribution of the genotype as it was observed for AAC and TP, while by contrast both the growing system and its interaction with the genotype did not provide significant contribution to the observed variation in red or yellow/orange carotenoids (Table 2). Therefore, these results suggest that the growing system (organic vs. conventional) had no mean effect on the level of carotenoids, and that the opportunity for exploiting the interaction genotype × growing system is much lower for these compounds than that for AAC and TP.

### Ascorbic acid content (AAC)

A broad range of variation for AAC in both growing systems and ripening stages was found in this experiment (Table 3). Thus, mean values of studied accessions at the unripe stage ranged from 14 to 121 mg·100 g^-1^ of fresh weight for Doux Long des Landes and Petit Marsellaise, respectively, in organic conditions, while values were comprised between 10 to 93 mg·100 g^-1^ for Jalapa F1 and BOL-144 under conventional conditions (Table 3). At the fully ripe stage AAC values increased considerably with values in organic ranging from 48 to 208 mg·100 g^-1^ in Ají dulce and Mojo Palmero, respectively, and from 48 to 196 mg·100 g^-1^ in Serrano and Petit Marsellaise, respectively, in conventional conditions (Table 3). In addition to these wide ranges of variation, it should be noted that the observed AAC values could be considered very high in general. Thus, the recommended daily intake of vitamin C in adults is 60–70 mg [35] and consequently many of our materials may satisfy this threshold with an intake of 50–100 g of fresh fully ripe fruit. Even several accessions at the unripe stage, where the content was clearly lower, also showed very high levels (≥ 60 mg·100 g^-1^). All these findings demonstrate the richness of *Capsicum* fruits in this antioxidant. In fact, other vegetables like tomato (25 mg·100 g^-1^), kiwifruit and oranges (50 mg·100 g^-1^) have in general levels considerably lower than those from our collection [41]. Moreover, in comparison to previous reports of peppers in organic [32,42,43], we observed that our ranges of variation were considerably higher, supporting the advantage of using wide collections to assess the real performance of a crop under different growing conditions, which also offers the opportunity of selecting very high-vitamin C genotypes. Moreover, it has always believed that fleshy, sweet, high water-content bell peppers have, in general, lower levels of ascorbic acid than hot/spicy thinner-mesocarp genotypes [41]. However, our results indicate that such acceptance is not so obvious. In fact, in most cases, within each ripening stage and growing condition, the group of bell peppers showed similar or higher AAC average than the other groups with smaller and thin-flesh fruits (other *C*. *annuum* and other Capsicums) (Table 3).

**Table 3:** Average content in ascorbic acid (AAC, mg·100 g^-1^ fresh weight) estimated in the unripe and fully ripe fruits from the peppers studied, evaluated under organic and conventional conditions and regression coefficient (β) based on environmental means.

Genotype	AAC unripe stage (mg·100 g^-1^)			AAC fully ripe stage (mg·100 g^-1^)		
	Organic	Conventional	β	Organic	Conventional	β
*C*. *annuum*						
Bell peppers						
Bierzo	25.1	43.7	-157.55***	120.9	102.3	1.04***
CW Aguila F_1_	39.7	59.0	-163.66***	150.6	142.1	0.48*
CW breeding line	39.9	84.2	-376.35***	150.8	112.7	2.14***
Cuneo	27.5	45.0	-148.77***	131.5	111.4	1.12***
Najerano	24.7	17.4	62.31*	163.8	148.5	0.86***
Pimiento Valenciano	18.2	52.3	-289.68***	139.4	106.0	1.87***
Mean	29.2	50.3		142.8	120.5	
Other *C*. *annuum*						
Ancho	25.1	76.4	-435.57***	126.3	188.9	-3.51***
Arnoia	52.9	30.5	189.83***	117.2	92.3	1.40***
Berbere	26.4	57.0	-259.59***	173.1	188.0	-0.83***
Bola	55.2	87.0	-270.46***	129.9	118.9	0.62**
Chile de Arbol	20.0	25.3	-44.17*	143.2	98.8	2.49***
Chimayo	76.4	22.4	458.42***	182.6	148.8	1.89***
Di Senise	65.1	54.1	93.63**	124.9	117.2	0.43^NS^
Doux Long des Landes	14.0	31.0	-144.39***	121.1	119.1	0.12^NS^
Espelette	26.7	37.7	-93.75**	192.2	151.1	2.30***
Gernika	72.9	92.6	-167.99***	172.3	147.3	1.40***
Guindilla Ibarra	29.8	26.4	28.83^NS^	123.2	95.7	1.54***
Jalapa F1	23.9	10.2	116.07***	93.9	88.9	0.28^NS^
Jalapeno Candelaria	16.7	32.4	-132.79***	66.2	66.2	0.00^NS^
Jalapeno Espinalteco	18.5	22.7	-36.16^NS^	70.8	66.3	0.25^NS^
Mojo Palmero	76.2	28.0	409.07***	208.0	129.0	4.42***
Numex 6–4	65.3	19.8	385.82***	159.8	122.2	2.11***
Numex Big Jim	57.4	44.2	112.12***	175.8	147.8	1.57***
Numex Conquistador	52.6	19.3	282.18***	151.2	118.8	1.82***
Padron	106.9	44.8	527.09***	155.4	102.9	2.94***
Pasilla	35.7	46.2	-89.05**	169.4	142.6	1.50***
Petit Marsellais	121.1	93.5	234.14***	178.1	196.2	-1.01***
Piquillo	71.0	52.4	158.64***	89.6	95.6	-0.33^NS^
Serrano Criollo	63.4	25.2	323.88***	179.2	110.8	3.83***
Serrano	32.0	36.0	-34.43^NS^	89.3	48.0	2.31***
Mean	49.5	42.3		141.4	120.9	
*C*. *annuum* mean	45.4	43.9		141.7	120.8	
Other *Capsicum* species						
Aji dulce	24.2	51.0	-227.05***	47.9	68.7	-1.16***
BOL-37R	54.5	82.3	-235.45***	94.1	88.1	0.34^NS^
BOL-58	31.2	28.2	25.54^NS^	68.5	74.6	-0.34^NS^
BOL-144	26.1	93.5	-572.11***	112.4	123.3	-0.61*
ECU-973	19.0	40.5	-182.07***	64.1	79.6	-0.87***
ECU-994	46.6	35.4	94.89***	104.1	117.1	-0.73**
PI-152225	36.7	17.3	164.61***	111.9	119.1	-0.40^NS^
Other *Capsicum* mean	34.0	49.7		86.2	95.8	
Total mean	45.6 a^a^	45.5 a		134.5 b	116.7 a	
Environmental effect	0.06	-0.06		8.93	-8.93	
Standard error	5.39	5.97		8.59	9.48	

^NS^, *, ** and *** indicate not significant for a probability p >0.05 and significant for p <0.05, 0.01 and 0.001, respectively, according to the statistical F ratio.

^a^ Total means with different letters within each ripening stage indicate significant differences at p< 0.05.

Regarding the effect of the growing system, at the unripe stage we observed as a whole similar mean AAC values in both organic and conventional conditions, around 45 mg·100 g^-1^ (Table 3), which is in agreement with the nonsignificant contribution of this factor for AAC at the unripe stage detected by ANOVA (Table 2). Nevertheless, this lack of effect in the general mean must be explained in detail, as there was a significant contribution of the growing system but depending on its interaction with the genotype and groups of genotypes. Thus, remarkable differences were found comparing the three *Capsicum* groups, as well as the performance of individual accessions. Thus, the group of bell peppers *C*. *annuum* and other Capsicums showed average values higher in conventional conditions than in organic, while the contrary was true in the other *C*. *annuum* group (Table 3). Even more, depending on the growing system, a very variable response could be observed in the different genotypes, which confirmed the significant interaction genotype × growing system detected in the ANOVA for this ripening stage (Table 2). By contrast, a different behaviour was found at the fully ripe stage, where the total AAC mean was higher in organic than conventional (135 vs. 117 mg·100g^-1^), and the same was found in *C*. *annuum* (142 vs. 121 mg·100g^-1^ in bell peppers and other *C*. *annuum*) (Table 3). Also, most studied accessions showed higher values in organic and only accessions from the other *Capsicum* species showed an erratic performance (Table 3). All these results were reflected in a high contribution of the genotype×growing system interaction to the variation in AAC at fully ripe fruits (Table 2).

In this regard, despite ACC average levels at both growing systems were similar at the unripe stage, the considerable contribution of the genotype×environment interaction provided significant differences among cultivars from one growing system to the other. Thus, within the unripe stage and according to the regression coefficient, fifteen accessions showed significantly higher AAC values under organic (β significantly > 0), being Chimayo, Mojo Palmero, Padron, Petit Marsellaise and Piquillo the cultivars with the highest values and increases due to this growing system. By contrast, eighteen genotypes had a better response under conventional practices (β significantly < 0), of which Bola, BOL-37R, BOL-144, California breeding line and Gernika were the ones with the best behaviour in these growing conditions. Finally, four accessions showed a stable behaviour between growing systems (β = 0). In the case of fully ripe fruits, where organic conditions provided on average higher ACC, most cultivars followed this trend and showed significant higher values under organic than under conventional practices, although accessions differed considerably in the magnitude of such differences, which was mainly due to the genotype × environment interaction. Thus, Chimayo, Espellete, Mojo Palmero, Numex Big Jim and Serrano Criollo showed the highest values in organic conditions and considerably higher than their values under conventional conditions (Table 3). Despite this general trend, the genotype × environment interaction also enabled a few accessions to have better AAC values under conventional practices like Ancho, Berbere and Petit Marsellais. Finally, several genotypes did not show significant differences between growing systems, like Jalapenos, Di Senise, Doux Long des Landes and Piquillo and some *C*. *baccatum* and *C*. *chinense* accessions, which could be considered stable under both systems (Table 3).

### Total phenolics (TP)

As it occurred in ascorbic acid, TP values were highly variable depending on the genotype and particularly growing conditions (Table 4). Regarding the genotype effect, at unripe stage and considering organic conditions, TP values were comprised between 68 and 626 mg chlorogenic acid equivalents (c.a.)·100 g^-1^ fresh weight for California breeding line and BOL-144 respectively, and between 87 and 506 mg·100 g^-1^ for the same accessions, respectively, under conventional conditions (Table 4). Similarly to AAC, TP values increased in most cases with ripening. Thus, at fully ripe stage, values ranged in organic between 109 and 367 mg·100 g^-1^ for Aji Dulce and Najerano, respectively, and between 96 and 438 mg·100 g^-1^ in conventional for Jalapeno Candelaria and BOL-144 respectively (Table 4). In comparison, these TP values in both unripe and fully ripe stages were considerably higher than those from other reference species as tomato, broccoli or banana, with average contents of 68 and 88 mg catechol equivalents·100 g^-1^ fresh weight, and 36 mg gallic acid equivalents·100 g^-1^ fresh weight, respectively [44,45], are in the lower limits of our TP intervals and therefore reinforces the idea of peppers as fruits very rich in antioxidants.

**Table 4:** Average content in total phenolics content (TP, mg chlorogenic acid equivalents·100 g^-1^ fresh weight) estimated in the unripe and fully ripe fruits from the peppers studied, evaluated under organic and conventional conditions and regression coefficient (β) based on environmental means.

Genotype	TP unripe stage (mg·100 g^-1^)			TP fully ripe stage (mg·100 g^-1^)		
	Organic	Conventional	β	Organic	Conventional	β
*C*. *annuum*						
Bell peppers						
Bierzo	78.3	114.2	1.43***	169.6	179.8	-0.38^NS^
CW Aguila F_1_	76.4	144.2	2.71***	159.0	120.7	1.44***
CW breeding line	67.7	86.6	0.76***	169.8	103.5	2.50***
Cuneo	71.5	94.0	0.90***	163.1	172.1	-0.34^NS^
Najerano	72.0	89.2	0.69***	366.9	106.1	9.83***
Pimiento Valenciano	110.7	151.6	1.63***	129.3	122.5	0.26^NS^
Mean	79.5	113.3		193.0	134.1	
Other *C*. *annuum*						
Ancho	128.6	209.9	3.25***	231.3	220.3	0.41^NS^
Arnoia	97.1	122.9	1.03***	113.3	116.3	-0.11^NS^
Berbere	80.8	243.3	6.49***	187.2	232.1	-1.69***
Bola	99.3	162.9	2.54***	190.5	195.5	-0.19^NS^
Chile de Arbol	119.6	154.5	1.39***	251.8	165.5	3.25***
Chimayo	102.2	119.3	0.68***	271.2	153.8	4.42***
Di Senise	101.2	117.6	0.66***	238.7	188.8	1.88***
Doux Long des Landes	130.6	211.7	3.24***	251.1	210.7	1.53***
Espelette	111.7	185.6	2.95***	257.5	179.1	2.96***
Gernika	130.2	215.0	3.39***	274.0	390.3	-4.39***
Guindilla Ibarra	136.7	136.7	0.00^NS^	295.8	241.1	2.06***
Jalapa F1	85.7	89.8	0.16^NS^	215.7	110.1	3.98***
Jalapeno Candelaria	229.3	89.4	-5.59***	115.6	96.1	0.74**
Jalapeno Espinalteco	113.0	126.8	0.55**	121.1	101.3	0.74**
Mojo Palmero	134.7	122.6	-0.48**	290.6	247.4	1.63***
Numex 6–4	99.7	96.9	-0.11^NS^	351.0	162.6	7.10***
Numex Big Jim	84.0	93.1	0.36*	281.6	192.5	3.36***
Numex Conquistador	86.3	113.5	1.09***	183.9	144.1	1.50***
Padron	190.0	153.0	-1.48***	246.5	248.1	-0.06^NS^
Pasilla	151.2	192.7	1.66***	286.3	269.1	0.65*
Petit Marsellais	148.1	139.1	-0.36*	355.7	217.6	5.21***
Piquillo	94.4	98.8	0.18^NS^	205.5	173.4	1.21***
Serrano Criollo	159.4	170.5	0.45*	176.4	237.2	-2.29***
Serrano	128.4	168.4	1.60***	232.3	236.9	-0.17^NS^
Mean	122.6	147.2		234.4	197.1	
*C*. *annuum* mean	114.0	140.5		226.1	184.5	
Other *Capsicum* species						
Aji dulce	148.6	176.0	1.10***	108.9	190.3	-3.07***
BOL-37R	257.1	242.8	-0.57**	248.7	195.4	2.01***
BOL-58	108.4	104.5	-0.16^NS^	302.6	283.2	0.73**
BOL-144	626.3	506.1	-4.81***	339.3	438.1	-3.73***
ECU-973	258.5	214.2	-1.77***	142.3	141.1	0.05^NS^
ECU-994	164.6	180.6	0.64***	198.9	219.1	-0.76**
PI-152225	106.1	227.0	4.83***	265.9	170.7	3.59***
Other *Capsicum* mean	238.5	235.9		229.5	234.0	
Total mean	125.5 a^1^	150.5 b		232.1b	205.6 a	
Environmental effect	-12.51	12.51		108.9	190.3	
Standard error	9.59	11.14		24.54	24.60	

^NS^, *, ** and *** indicate not significant for a probability p >0.05 and significant for p <0.05, 0.01 and 0.001, respectively, according to the statistical F ratio.

^1^ Total means with different letters within each ripening stage indicate significant differences at p< 0.05.

The growing system had a significant effect in the phenolic values for both unripe and fully ripe stage as indicated by the ANOVA (Tables 2 and 4). Thus, at the unripe stage, total mean was higher in conventional conditions than organic, with values of 151 and 126 mg·100 g^-1^, respectively, and the same trend was found in *C*. *annuum* (80 and 113 mg·100 g^-1^ in organic and conventional in bell peppers; 123 and 147 mg·100 g^-1^ in other *C*. *annuum*), while means were similar in other *Capsicum* species (about 240 mg·100 g^-1^) (Table 4). Moreover, considering further the genotype×environment interaction, in terms of both the magnitude of the increase/decrease of TP and the sense of this behavior and according to the regression study, twenty-five accessions showed higher TP values in the conventional system, being Berbere, Doux Long des Landes, Gernika and several genotypes from other *Capsicum* species, particularly PI-152225, the accessions with the highest values (200–500 mg·100 g^-1^), while seven accessions had higher TP values under organic cultivation, being Jalapeno Candelaria, Padrón, BOL-37R, BOL-144 and ECU-973 the ones with the highest values (190–600 mg·100 g^-1^), and five accessions were stable (Table 4).

Contrary to these results, fully ripe fruits showed, on the whole, higher TP levels under organic conditions than under conventional conditions (total means of 232 vs 206 mg·100 g^-1^, respectively) and the same was found in most cultivars, particularly in *C*. *annuum* (226 vs 185 mg·100 g^-1^) (Table 4), similarly to AAC. Nevertheless, other *Capsicum* species showed a less consistent trend, highly depending on the accession, and means from organic and conventional conditions were very similar (about 230 mg·100 g^-1^). This fact confirmed again the high contribution of the ripening stage and their interactions to the observed variation in TP, as well as in AAC (Table 2). Regarding the individual behavior of the different accessions, twenty-two accessions had better behaviour in organic, where BOL-58, Guindilla Ibarra, Numex 6–4, Najerano and Petit Marsellaise showed the highest values (300–360 mg·100 g^-1^), while only six accessions showed a better performance in conventional conditions, being Gernika and BOL-144 the most outstanding materials (>300 mg·100^−1^), and nine accessions were stable (Table 4).

#### Total red carotenoids and yellow/orange carotenoids (TC_R_,TC_Y/O_)

The range of variation in carotenoids in fully ripe *Capsicum* fruits was very wide, similar to that observed in AAC and TP, and it confirmed the high contribution of the genotype factor to the observed variation (Tables 2 and 5). Organic conditions registered values of red carotenoids (TC_R_) comprised between 0 and 257 mg·100 g^-1^ of fresh matter from yellow-fruited cultivars like Cuneo or Petit Marsellais and brownish-fruited cultivar Pasilla, respectively, and TC_Y/O_ between 2 and 147 mg·100 g^-1^ for Jalapeno Espinalteco and BOL-58 respectively (Table 5). Within the conventional growing system, TC_R_ values ranged again between 0 and 324 mg·100 g^-1^ in the same genotypes than organic system, and TC_Y/O_ were comprised between 1 and 166 mg·100 g^-1^ for Jalapeno Espinalteco and Gernika respectively (Table 5). These values of total carotenoids were relatively high in comparison to other vegetables and fruits like broccoli, tomato, carrot, apricot or squash (around 5, 7, 15, 17 and 41 mg·100 g^-1^, respectively) [46–48] and makes clear that *Capsicum* pods are a rich source of this bioactive compounds. In addition, by contrast to AAC and TP results, bell peppers had, in general, considerably lower amounts of carotenoids than the other *C*. *annuum* and other *Capsicum* species. Thus, the average mean of bell peppers was 18 and 11 mg·100 g-1 in TC_R_ and TC_Y/O_, respectively, and only Bierzo (65 and 30 mg·100 g^-1^) reached the usual levels of the other groups, which showed a thin mesocarp and were less fleshy than bell peppers, with the only exception of Arnoia and Jalapeno types. Such results can be explained by the high correlation between carotenoids and dry matter content in peppers [41].

**Table 5:** Average content in total red carotenoids and yellow/orange carotenoids (TC_R_ and TC_Y/O_, mg·100 g^-1^ fresh weight) estimated in the unripe and fully ripe fruits from the peppers studied, evaluated under organic and conventional conditions and regression coefficient (β) based on environmental means.

Genotype	TC_R_ fully ripe stage (mg·100 g^-1^)			TC_O/Y_ fully ripe stage (mg·100 g^-1^)		
	Organic	Conventional	β	Organic	Conventional	β
*C*. *annuum*						
Bell peppers						
Bierzo	63.8	65.8	0.20^NS^	33.0	27.8	-15.29^NS^
CW Aguila F_1_	5.1	7.0	0.18^NS^	1.7	2.6	2.84^NS^
CW breeding line	11.8	8.2	-0.33^NS^	3.5	3.2	-0.82^NS^
Cuneo	0.0	0.0	0.00^NS^	16.5	17.1	1.96^NS^
Najerano	17.1	5.3	-1.11^NS^	7.9	2.7	-15.59^NS^
Pimiento Valenciano	7.8	17.6	0.93^NS^	2.7	7.2	13.55^NS^
Mean	17.6	17.5		10.9	10.1	
Other *C*. *annuum*						
Ancho	22.1	30.9	0.83^NS^	12.9	24.2	33.74*
Arnoia	6.7	8.2	0.14^NS^	3.2	5.9	7.98^NS^
Berbere	29.5	22.7	-0.64^NS^	20.8	12.6	-24.63^NS^
Bola	136.5	161.3	2.33***	71.5	67.2	-12.89^NS^
Chile de Arbol	31.9	34.7	0.27^NS^	16.8	7.9	-26.69^NS^
Chimayo	25.6	32.4	0.63^NS^	12.6	18.7	18.45^NS^
Di Senise	114.9	124.4	0.90^NS^	80.7	74.5	-18.68^NS^
Doux Long des Landes	105.6	109.5	0.37^NS^	77.1	78.1	3.04^NS^
Espelette	127.7	195.0	6.33***	106.8	102.2	-13.67^NS^
Gernika	179.9	263.3	7.85***	137.5	165.9	85.01***
Guindilla Ibarra	211.5	144.4	-6.31***	119.5	103.6	-47.58***
Jalapa F1	7.1	24.9	1.67**	2.4	17.4	44.86**
Jalapeno Candelaria	5.1	7.5	0.22^NS^	2.3	5.3	8.86^NS^
Jalapeno Espinalteco	8.0	2.8	-0.49^NS^	2.3	0.7	-4.87^NS^
Mojo Palmero	131.6	189.0	5.41***	104.7	128.3	70.65***
Numex 6–4	18.3	21.7	0.32^NS^	12.3	14.5	6.50^NS^
Numex Big Jim	110.3	112.9	0.24^NS^	76.8	65.5	-33.82*
Numex Conquistador	28.7	27.2	-0.14^NS^	13.7	9.3	-13.14^NS^
Padron	164.4	181.3	1.59*	102.5	113.5	-14.14^NS^
Pasilla	257.0	324.3	6.34***	93.3	76.0	60.39***
Petit Marsellais	0.0	0.0	0.00^NS^	5.1	6.4	3.92^NS^
Piquillo	138.1	136.9	-0.11^NS^	72.1	71.6	-1.64^NS^
Serrano Criollo	25.4	33.9	0.79^NS^	10.9	13.8	8.43^NS^
Serrano	102.5	111.7	0.87^NS^	49.6	47.3	-6.77^NS^
Mean	82.9	95.9		50.3	52.2	
*C*. *annuum* mean	69.9	80.3		42.4	43.8	
Other *Capsicum* species						
Aji dulce	11.8	10.6	-0.11^NS^	6.8	5.6	-3.49^NS^
BOL-37R	23.8	19.2	-0.43^NS^	13.5	11.4	-6.49^NS^
BOL-58	181.8	192.8	1.03^NS^	146.8	141.3	-16.24^NS^
BOL-144	30.5	33.7	0.31^NS^	26.1	20.5	-16.65^NS^
ECU-973	14.3	21.3	0.66^NS^	7.5	11.6	12.10^NS^
ECU-994	144.6	127.6	-1.60*	79.8	70.7	-27.24^NS^
PI-152225	99.1	41.7	-5.41***	24.8	16.4	-24.92^NS^
Other *Capsicum* mean	72.3	63.8		43.6	39.7	
Total mean	92.8 a^a^	103.4 a		57.6 a	57.9 a	
Environmental effect	-5.31	5.31		-0.17	0.17	
Standard error	21.99	22.24		11.11	11.23	

^NS^, *, ** and *** indicate not significant for a probability p >0.05 and significant for p <0.05, 0.01 and 0.001, respectively, according to the statistical F ratio.

^a^ Total means with different letters within each ripening stage indicate significant differences at p< 0.05.

Moreover, the level in carotenoids showed by several accessions was very interesting. Thus, Pasilla was the cultivar with the highest values TC_R_ and very high TC_Y/O_, regardless the growing system (Table 5). This fact suggests paradoxically that the chlorophyll retainer (*cl*) mutation, characterized by inhibition of chlorophyll degradation during fruit ripening and causing brown fruits (due to the combination of chlorophylls and red carotenoids) [49], also provides high levels of carotenoid pigments [29]. In addition, other peppers like Bola, Espelette or Mojo Palmero also showed very high TC_R_ levels (Table 5), which explain why they were mainly selected to be used as food colorants in different food preparations (e.g. ground powder, sauces, spicy pork sausages and chorizo) [8]. Moreover, it should be also pointed out the performance of cultivars like Gernika, Guindilla de Ibarra or Padron, which have been selected for consumption at the unripe stage (i.e. roasted or pickled at very early unripe fruit stage), had very high average measures of TC_R_, suggesting that their use at the fully ripe stage can be promoted as, in fact, it has been partly started in the Basque Country, where they are called Chorizeros (to make chorizo) when fully ripe [8]. Finally, Di Senise, Numex Big Jim and Piquillo, despite used when fully ripe but not as food colorants they also showed high TC_R_ levels (>100 mg·100 g^-1^), as well as the exotic BOL-58 and ECU-994 (100–200 mg·100 g^-1^) (Table 5), indicating that materials not selected originally as food colorants can be also fully ripe selected for high red carotenoid content.

In terms of yellow/orange carotenoids, we found a high correlation with red carotenoids and therefore those accessions with the highest TC_R_ values mentioned before were also the ones with the highest TC_Y/O_ values (70–170 mg·100 g^-1^). These levels in yellow and red carotenoids (including β-carotene and β-cryptoxanthin) indicates that these accessions can provide high levels of provitamin-A as reported in previous works [29,31] while yellow-fruited accessions Cuneo and Petit Marsellais showed very low TC_Y/O_ values (Table 5). Such fact can be explained because red-fruited peppers contain both red and yellow/orange carotenoids, and the red pigments (capsanthin and capsorubin) are synthetized from yellow and orange precursors, feeding back the new synthesis of yellow/orange carotenoids [50].

Total average was similar in both growing systems for both TC_R_ and TC_Y/O_ confirming the nonsignificant effect of growing system detected in the ANOVA (Tables 2 and 5). Thus, TC_R_ total average was 93 and 103 mg.100 g^-1^ for organic and conventional systems respectively, and around 58 mg.100 g^-1^ for TC_Y/O_ in both systems, and the same was found considering the different groups of peppers (Table 5). Moreover, in comparison to AAC and TP very few accessions showed significant differences for TC_R_ and TC_Y/O_ between organic and conventional conditions, which explains the lack of significance of the genotype × growing system interaction (Table 2). Thus, for TC_R_ only three accessions showed higher levels in organic conditions compared to conventional (ECU-994, Guindilla Ibarra and PI-152225), while the contrary was found in seven accessions (Table 5). The behavior in TC_Y/O_ was similar with only two accessions significantly better under organic conditions (Guindilla Ibarra and Numex Big Jim) and five under conventional conditions (Ancho, Gernika, Jalapa F1, Mojo and Pasilla) (Table 5). This fact was previously reported by some authors [51,52], although other studies found significant differences due to growing conditions [32,34]. Moreover, it has been described that the content in carotenoids depended on genotype, soil type and grower-specific inputs as fertilizers and methods of pests control [53]. From our experiment, performed with a large diversity of varietal types, we can conclude that the factors that enhance in general AAC and TP under organic conditions do not have this effect in carotenoids. Probably the antioxidant nature of ascorbic acid and phenolics drives to increase their levels in plant tissues in response to free-radicals accumulation under more stressing-low input conditions of our organic trials. By contrast, the primary role of carotenoids as photoreceptors and photoprotectors did not provide any response.

### Ripening process in AAC and TP under organic and conventional practices

**Fig 1:**
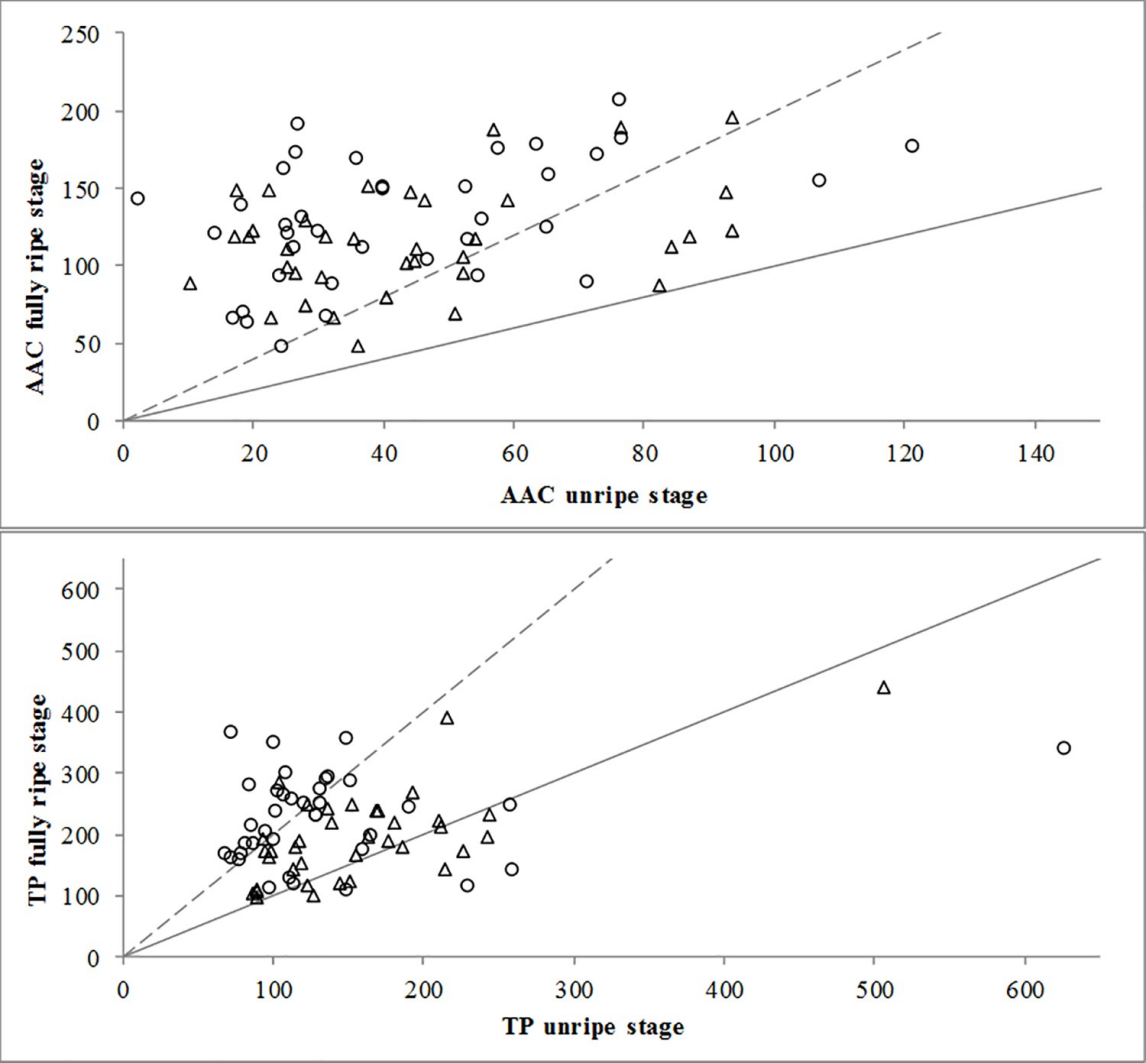
Comparative study of the content of ascorbic acid (AAC, upper, mg·100 g^-1^ fresh weight) and total phenolics (TP, bottom, mg·100 g^-1^ fresh weight), in organic (○) and conventional (Δ) growing systems. The oblique lines included in the graph represent slope 1 (continuous line, ratio fully ripe/unripe = 1, increase 0% with ripening) and slope 2 (discontinuous line, ratio TP fully ripe/TP unripe = 2, increase 100% with ripening).

Ripening process favoured the accumulation of AAC in fully ripe fruits. Thus, all the varieties showed higher AAC at fully ripe stage than at unripe stage (slope fully ripe/unripe > 1, Fig 1). These findings agree with reports from other authors [19,25,54], although the large number of accessions used in our study showed a wide variation in the magnitude of such increase, mainly depending on the genotype but also modified by growing conditions.

In this regard, some accessions showed an increase in AAC during ripening comprised between 1% and 100% (1< slope ≤ 2), although in most accessions the ripening process increased AAC more than 100% (slope > 2) (Fig 1). In these last cases, there was a range of increases with ripening and even some accessions showed levels at the fully ripe stage 4–8 fold from those at the unripe stage (Fig 1).

In comparison, organic conditions enabled higher increases in AAC than conventional conditions, and data from organic grouped mainly in the areas of higher slopes (Fig 1). This was in agreement with the results observed on Table 3, where AAC total means increased with ripening but at higher extent in organic, and also the increase of most accessions was higher under organic.

As observed for AAC, the accumulation of TP increased with the ripening process in general and in most genotypes (slope fully ripe/unripe ≥ 1, Fig 1), although this effect was not so consistent and even some accessions, five in organic and ten in conventional, had lower values at the fully ripe stage (Fig 1). Our results agree with a review of other reports. Thus, the accumulation of phenolic compounds with ripening has been described but also the decrease and even depending on the cultivars [55–57].

The studied accessions showed different magnitudes of TP increase with ripening, as genotype × ripening stage interaction showed in the ANOVA (Table 2) as can be observed in the range of slopes displayed in Fig 1. It should be also noted that the increase in TP with ripening could be modified by growing system conditions (i.e. growing system × ripening stage interaction, Table 2). In this sense, within the organic growing system eleven accessions had slopes comprised between 1 and 2 and twenty-one accessions had slopes between 2 and 5 (Fig 1). By contrast, within the conventional system twenty-four accessions showed slopes comprised between 1 and 2, while only three accessions had slopes ranged between 2 and 3 (Fig 1). Thus, similarly to AAC, the organic growing system appeared to increase the accumulation of TP with the ripening process at a higher extent than conventional conditions.

These results suggest that, in general, organic conditions may strengthen the increase of AAC with the ripening process in a more intense way than conventional conditions. In this regard, the different behaviour detected between unripe and fully ripe fruits in terms of TP and AAC should be considered. Thus, the levels of both antioxidants in fully ripe fruits were higher (in general and in most accessions) in organic cultivation, while the contrary was true in unripe fruits. These findings suggest that some factors present in organic conditions may increase the accumulation of antioxidants in the fruits of most *Capsicum* genotypes, but mostly in fully ripe fruits. Perhaps, fruits during ripening are more sensitive to factors from soils, like those from high fertilized conditions typical of conventional practices, in comparison to less fertilized (organic) conditions. In this regard, some studies [58–61] reported that high rates of specific mineral fertilization could lead to nutrient dilutions and interactions. Thus, conventional soil management could cause interactions that disturb the balance of soil nutrients, essential in the metabolic pathways of biosynthesis, which could be one of the reasons that leads to a lower accumulation of ascorbic acid and phenolics at the end of ripening.

### Potential selections of materials for organic cultivation

Based on the results of our trials, a potential selection of plant material for organic cultivation would be possible considering high mean levels of bioactive factor, coefficient of regression, and varietal groups, as well as each ripening stage due to the importance of this factor for the commercial use of fruits.

**Fig 2:**
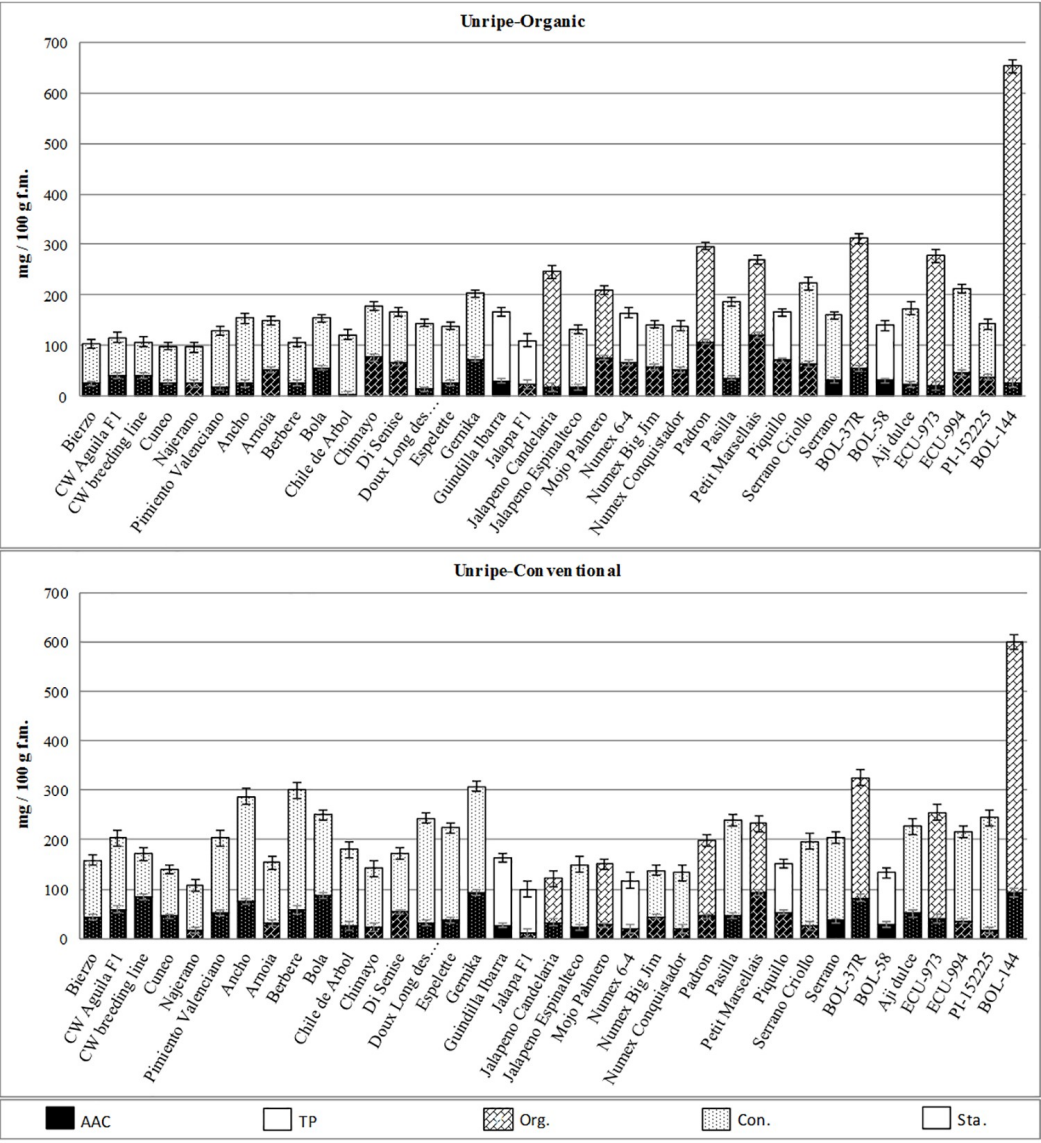
Comparison among accessions at the unripe stage considering ascorbic acid content (AAC) and total phenolics (TP) under organic (upper) and conventional (bottom) growing systems. Texture of columns, based on β parameter, indicates better response to organic (Org.), conventional (Con.) or stable behaviour (Sta.). Bars on the top of columns indicates standard error intervals.

Thus, within the unripe stage, *C*. *annuum* accessions such as Chimayo, Gernika, Jalapeno Candelaria, Mojo Palmero, Padrón, Pasilla, Petit Marsellais and Serrano Criollo would be the best options as their sum of AAC and TP reaches 200–300 mg·100 g^-1^, as well as Pimiento Valenciano among the fleshy bell peppers group (Fig 2). Also, many accessions from the exotic species like *C*. *chinense* Aji Dulce, ECU-973 and ECU-994, *C*. *baccatum* BOL-37R and particularly *C*. *frutescens* BOL-144 showed an outstanding performance, which offers an opportunity to these materials in new markets with the added value of their exotic appearance and levels of bioactive compounds under organic practices.

**Fig 3:**
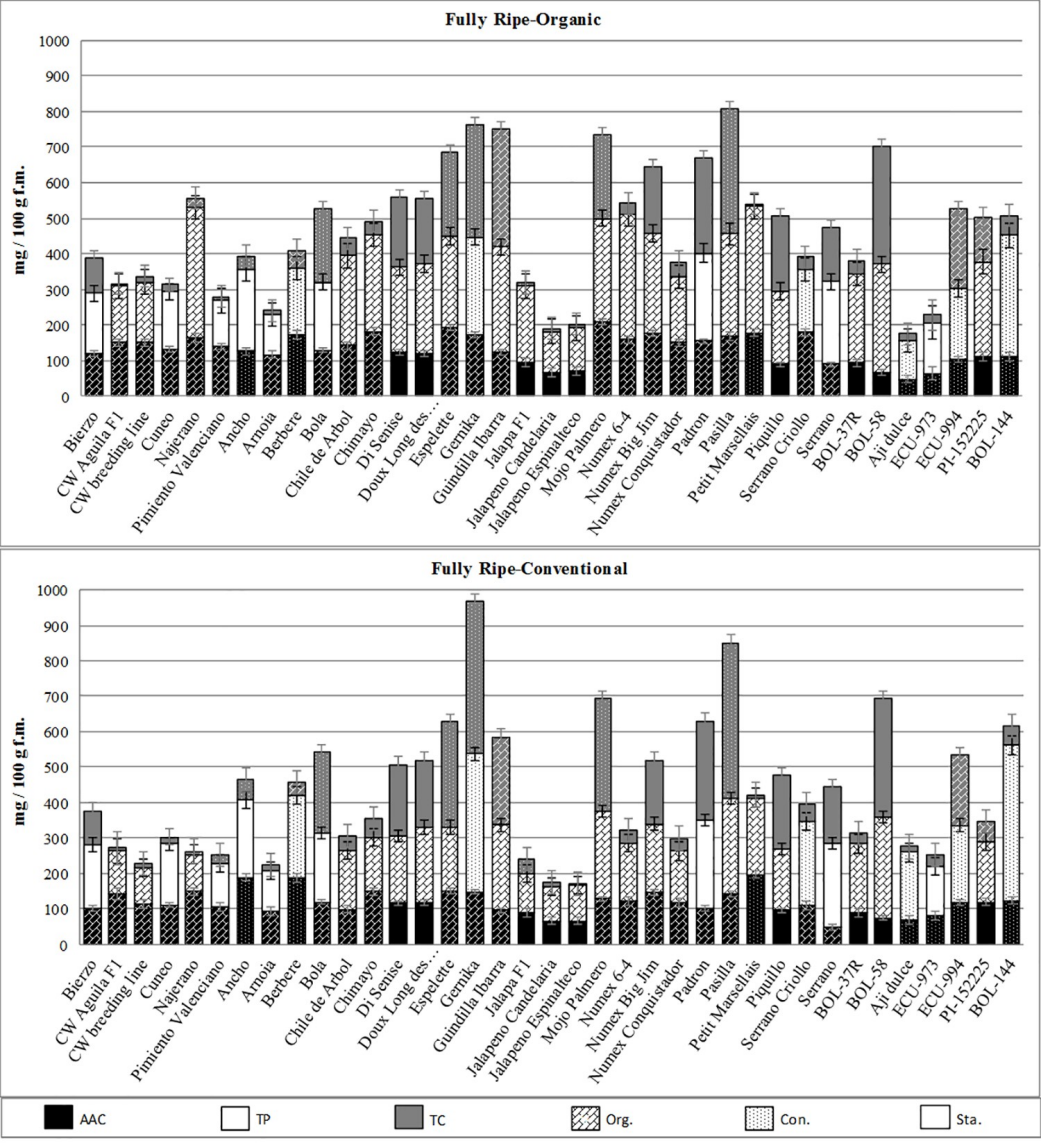
Comparison among accessions at the fully ripe stage considering ascorbic acid content (AAC), total phenolics (TP) and total carotenoids (TC_R_+TC_Y/O_) under organic (upper) and conventional (bottom) growing systems. Texture of columns, based on β parameter, indicates better response to organic (Org.), conventional (Con.) or stable behaviour (Sta.). Bars on the top of columns indicates standard error intervals.

Considering the fully ripe stage, several materials could be selected for organic cultivation. Thus, within *C*. *annuum* accessions like Bola, di Senise, Doux Long des Landes, Espelette, Gernika, Guindilla de Ibarra, Mojo Palmero, Numex 6–4, Numex Big Jim, Pasilla, Padron, Petit Marsellais and Piquillo, as well as Najerano, and even Bierzo, among the bell peppers group, offered very high total levels of bioactive compounds (500–800 mg.100 g^-1^) (Fig 3). Even more, *C*. *annuum* Chile de Arbol and Chimayo and *C*. *chinense* PI-152225 showed high levels of AAC and TP and Petit Marsellais, despite of very low carotenoids levels, is a good alternative among yellow/orange peppers. In addition, *C*. *baccatum* BOL-58, *C*. *chinense* ECU-994 and *C*. *frutescens* BOL-144 showed high total levels and could be selected among the exotic species for a niche of exotic products within the organic market, especially BOL-58 and ECU-994, because of their particular aroma and flavour at the ripe stage as reported by [62].

Finally, most of the mentioned materials also showed a high performance in conventional conditions, which suggests a wide adaptation to other growing conditions, including different organic production units (Figs 2 and 3). Even more, several materials like Chimayo, Disenise, Gernika, Guindilla Ibarra, Mojo Palmero, Padrón, Pasilla, Petit Marsellais, Piquillo, and the exotic *C*. *chinense* ECU-994 and *C*. *frutescens* BOL-144 offered remarkable levels of bioactive compounds regardless the ripening stage (Figs 2 and 3) and, therefore, its cultivation could be advised for producing high-quality fruits both unripe and fully ripe stages.

## Conclusions

In conclusion, our findings enabled to assess the impact of factors like the genotype, the ripening stage and their interactions on the response to organic cultivation (compared to conventional) for the levels of the main bioactive compounds in peppers, founded on a comprehensive varietal diversity of *Capsicum*. A wide range of variation was found among the studied accessions, regardless the ripening stage and growing conditions. Also, fully ripe fruits were found considerably richer than unripe fruits in the studied bioactive factors, with the only exception of very few accessions in phenolics; and organic cultivation provided, as a whole, higher levels of ascorbic acid and phenolics at this stage. Moreover, the remarkable genotype×environment interaction allowed the selection of several accessions with a remarkable performance under organic conditions at both ripening stages. By contrast the effects of growing conditions and the genotype×growing conditions were nil on the levels of carotenoids, whose variation was mainly due to the genotype factor. The results will be very helpful for breeders and scientists aimed at breeding high-added vegetables for adaptation to organic production systems.

## Supporting information

S1 Fig Diversity in fruit morphology in the studied collection of *Capsicum*: Accessions 1 (Bierzo) to 18 (Jalapa F1). (TIF)

S2 Fig Diversity in fruit morphology in the studied collection of *Capsicum*: Accessions 19 (Jalapeno Candelaria) to 37 (BOL-144). (TIF)

S1 Table Basic soil analyses of both plots used in the experiment right before transplanting. (DOCX)

S1 File Supporting data: Raw data from analyses. (XLSX)
