# Supplementary figures and images for: Response to organic cultivation of heirloom Capsicum peppers: Variation in the level of bioactive compounds and effect of ripening
Source: PLoS One. 2018 Nov 21;13(11):e0207888. doi: 10.1371/journal.pone.0207888 (PMC6249006; doi:10.1371/journal.pone.0207888)

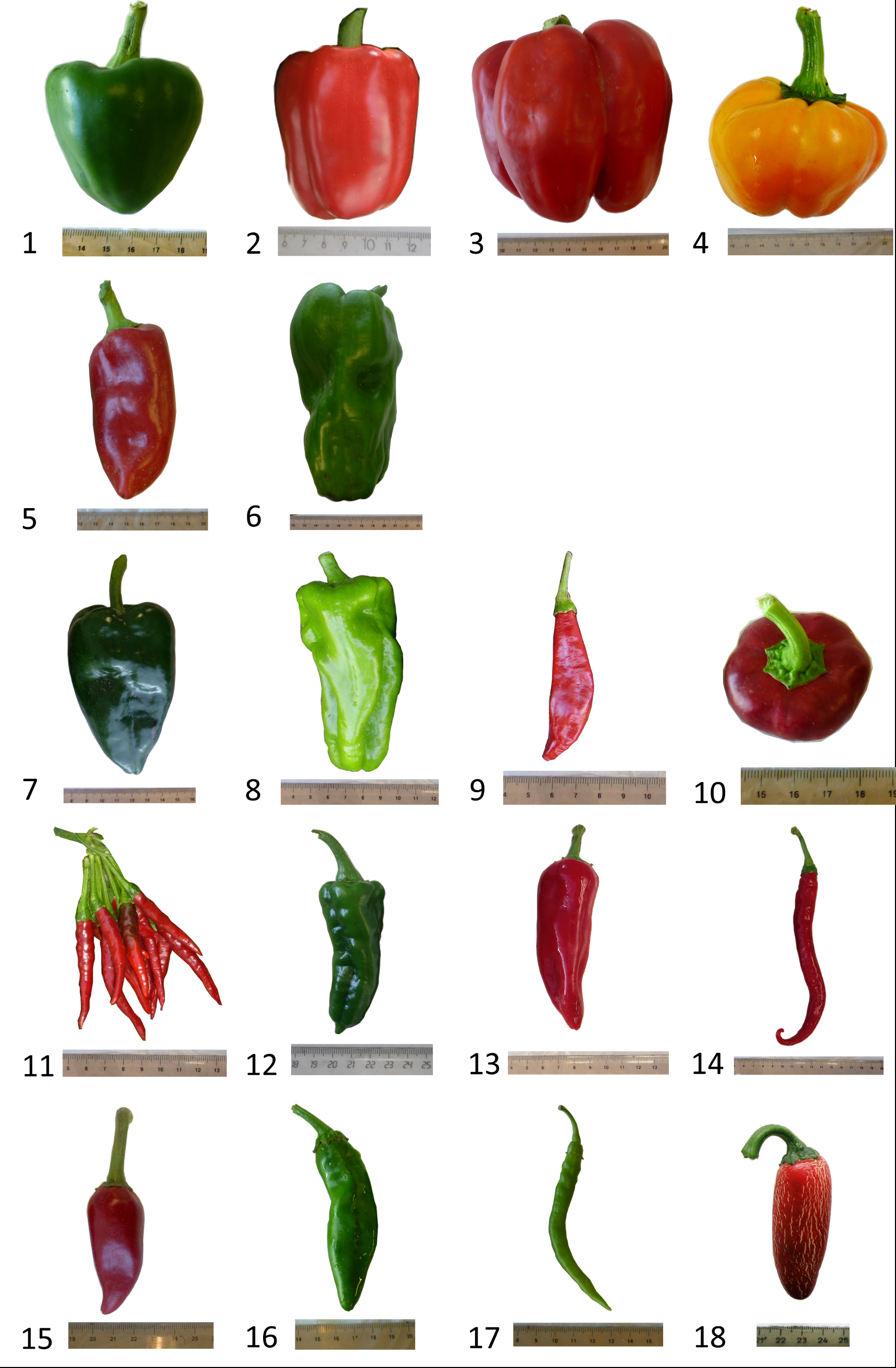

Supplement: S1 Fig — (TIF) [file pone.0207888.s001.tif]

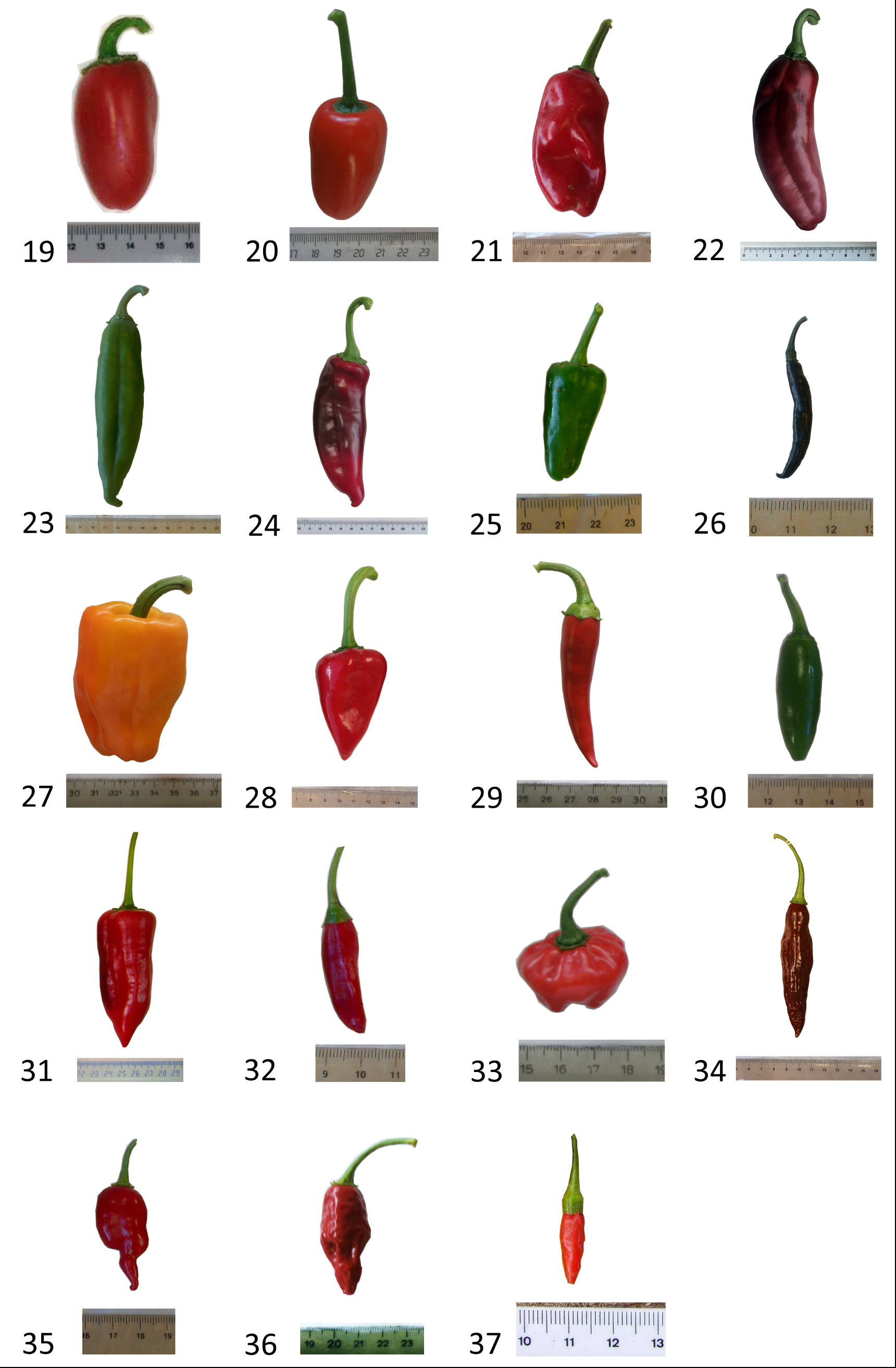

Supplement: S2 Fig — (TIF) [file pone.0207888.s002.tif]
