# Supplementary material for: Response to organic cultivation of heirloom Capsicum peppers: Variation in the level of bioactive compounds and effect of ripening
Source: PLoS One. 2018 Nov 21;13(11):e0207888. doi: 10.1371/journal.pone.0207888 (PMC6249006; doi:10.1371/journal.pone.0207888)
Supplement: S1 Table — (DOCX) [file pone.0207888.s003.docx]

S1 Table. Basic soil analyses of both plots used in the experiment right before transplanting.

| Parameter | Organic plot | Conventional plot |
| --- | --- | --- |
| Soil texture | Sandy-clay loam | Sandy-clay loam |
| Available water capacity (% d.w.) | 11.94 | 11.50 |
| pH (H_2_O-based) | 8.14 | 8.21 |
| EC (dS/cm) | 0.363 | 0.341 |
| CaCO_3_ (% d.w.) | 11.88 | 11.11 |
| N total (% d.w.) | 0.10 | 0.10 |
| Organic matter (% d.w.) | 2.49 | 2.05 |
| C/N ratio | 11.45 | 12.29 |
| P assimilable (ppm) | 92.06 | 92.30 |
| K (ppm) | 694 | 714 |
| Ca (ppm) | 4851 | 4002 |
| Mg (ppm) | 451 | 446 |
